# Risk of epilepsy after traumatic brain injury: a nationwide Norwegian matched cohort study

**DOI:** 10.3389/fneur.2024.1411692

**Published:** 2024-06-05

**Authors:** Hild Flatmark Sødal, Trond Nordseth, Anders Johan Orland Rasmussen, Leiv Arne Rosseland, Jo Steinson Stenehjem, Jon Michael Gran, Eirik Helseth, Erik Taubøll

**Affiliations:** ^1^Institute of Clinical Medicine, Faculty of Medicine, University of Oslo, Oslo, Norway; ^2^ERGO – Epilepsy Research Group of Oslo, Department of Neurology, Oslo University Hospital, Oslo, Norway; ^3^Department of Research and Development, Division of Emergencies and Critical Care, Oslo University Hospital, Oslo, Norway; ^4^Department of Anesthesia and Intensive Care Medicine, St. Olav Hospital, Trondheim, Norway; ^5^Department of Anaesthesia, Innlandet Hospital Trust, Hamar, Norway; ^6^Oslo Centre for Biostatistics and Epidemiology, Department of Biostatistics, University of Oslo, Oslo, Norway; ^7^Department of Research, Cancer Registry of Norway, Oslo, Norway; ^8^Department of Neurosurgery, Oslo University Hospital, Oslo, Norway

**Keywords:** post-traumatic epilepsy, traumatic brain injury, incidence, epidemiology, abbreviated injury scale

## Abstract

**Background:**

Post-traumatic epilepsy (PTE) is a well-known complication of traumatic brain injury (TBI). Although several risk factors have been identified, prediction of PTE is difficult. Changing demographics and advances in TBI treatment may affect the risk of PTE. Our aim was to provide an up-to-date estimate of the incidence of PTE by linking multiple nationwide registers.

**Methods:**

Patients with TBI admitted to hospital 2015–2018 were identified in the Norwegian Trauma Registry and matched to trauma-free controls on sex and birth year according to a matched cohort design. They were followed up for epilepsy in nationwide registers 2015–2020. Cumulative incidence of epilepsy in TBI patients and controls was estimated taking competing risks into account. Analyses stratified by the Abbreviated Injury Scale (AIS) severity score, Glasgow Coma Scale score and age were conducted for the TBI group. Occurrence of PTE in different injury types was visualized using UpSet plots.

**Results:**

In total, 8,660 patients and 84,024 controls were included in the study. Of the patients, 3,029 (35%) had moderate to severe TBI. The cumulative incidence of epilepsy in the TBI group was 3.1% (95% Confidence Interval [CI] 2.8–3.5%) after 2 years and 4.0% (3.6–4.5%) after 5 years. Corresponding cumulative incidences in the control group were 0.2% (95% CI 0.2–0.3%) and 0.5% (0.5–0.6%). The highest incidence was observed in patients with severe TBI according to AIS (11.8% [95% CI 9.7–14.4%] after 2 years and 13.2% [10.8–16.0%] after 5 years) and in patients >40 years of age.

**Conclusion:**

Patients with TBI have significantly higher risk of developing epilepsy compared to population controls. However, PTE incidence following moderate–severe TBI was notably lower than what has been reported in several previously published studies.

## 1 Introduction

Traumatic brain injury (TBI) is a major health problem and an important contributor to morbidity and mortality worldwide (1). Epileptic seizures may occur in the acute phase following injury, or after a latency period that may last for several years after TBI (2–4). A single seizure occurring more than 1 week after TBI is associated with a high risk of recurrent seizures and qualify for the diagnosis of post-traumatic epilepsy (PTE) (5, 6).

PTE is associated with poor functional outcomes (7, 8), and imposes additional personal and societal challenges following TBI. Despite efforts to discover effective prophylactic therapies, no such treatment is currently available. Promising results with novel therapies and repurposed drugs targeting mechanisms that are involved in epileptogenesis, brings hope for preventive treatment to patients at high risk of PTE (9, 10). For future clinical trials on potential anti-epileptogenic treatment to be successful, we need to be able to reliably identify high-risk patients that may benefit from this type of intervention.

The pathophysiologic processes leading to development of epilepsy in the injured brain are not completely understood, and the heterogeneity of TBI may impede our ability to identify subgroups of patients at high risk. The severity of TBI, commonly classified according to the initial Glasgow Coma Scale (GCS) score (11), is considered one of the main risk factors for PTE. Penetrating injuries and abnormal neuroimaging findings have been associated with an increased risk of PTE (12, 13), as have also other factors like age, sex and early post-traumatic seizures (14, 15).

Over the last decades, the demographics and injury characteristics of TBI have changed (16, 17). This, as well as advancements in TBI management may affect the incidence of PTE. According to a recent study on patients with severe TBI, the 5-year cumulative incidence of PTE was found to be as high as 25%, which may indicate a rising incidence over time (5). Up to date knowledge about the occurrence of PTE is essential for optimal treatment and care for the heterogeneous group of patients with TBI. It is also important when planning future clinical trials. Our aim was to provide a contemporary assessment of the incidence of PTE by linking multiple comprehensive nationwide registers.

## 2 Materials and methods

### 2.1 Study design and data sources

This is a matched cohort study conducted on all patients registered in the Norwegian Trauma Registry (NTR) between 1st of January 2015 and 31st of December 2018 matched to trauma-free controls from the Norwegian general population.

The Norwegian trauma system is publicly funded and includes 34 acute care trauma hospitals and four regional trauma centers. Since 2015, it has been mandatory for all hospitals receiving trauma patients to register these in the NTR, ensuring a nationwide coverage of >90% (18). Included in the registry are patients admitted through trauma team activation or who have a New Injury Severity Score > 12, head injury with Abbreviated Injury Scale (AIS) ≥3 or a penetrating injury to head, neck, torso, or proximal extremities. The NTR dataset is based on the Utstein template for uniform reporting of data following major trauma (19), and each injury observed has been coded according to the Abbreviated Injury Scale (AIS) 2005, update 2008 (20). Data collection is performed by certified registrars, ensuring a high degree of completeness and validity (21). All patients are informed by mail and given the option to be excluded from registry.

This study is part of the Injury Prevention and Outcomes following Trauma project, where a comprehensive research database has been established by coupling NTR-data to several independent, national registries using the Norwegian social security number (22). For each of the trauma cases in NTR, 10 controls have been drawn from the National Population Register to represent the Norwegian general population, according to risk-set sampling with replacement for a nested case–control (NCC) design. Controls had to be alive, resident in Norway and trauma-free at the injury date of the case (index date) and were matched to each case on sex and birth year. Data from the Norwegian Patient Registry (NPR), the Norwegian Prescription Database (NorPD) and Statistics Norway have been provided for the period 2014–2020, to ensure a one-year observational period before the trauma, and at least 2 years of follow-up. The NPR holds information on all specialized healthcare contacts including outpatient visits and admissions to hospitals and rehabilitation institutions. This includes diagnoses according to the International Classification of Diseases, Tenth Revision (ICD-10). The NorPD contains information on all dispensed prescription drugs from Norwegian pharmacies. The data flow and exclusions from the NTR cohort, sampling of controls and linkage of national registries and databases have been described by Stenehjem et al. (22).

### 2.2 Study population

We identified patients with TBI from the NTR cohort based on selected AIS codes, specified in Supplementary Table 1. AIS is an anatomical injury severity scoring system that describes injuries and ranks the severity from 1 to 6, where 6 represents a lethal injury (20). We included all AIS codes for intracranial injury and skeletal injury of the cranium, except codes describing lesions in scalp, nerves, vessels, cerebellum, brain stem and pituitary gland. Only patients with at least one AIS code consistent with TBI in the first occurring trauma were included.

For the present study, the controls sampled for the NCC study were reused in a matched cohort setting to estimate incidence of epilepsy both among patients with TBI and among the controls. Sampling of NCC-controls with replacement resulted in some individuals, by design, acting as controls for more than one case. When reusing NCC-controls in the present matched cohort study, we kept only one record per individual, resulting in less than 10 controls per TBI patient, representing a sample of the general population with similar distribution of age and sex as the patients.

**Figure 1:**
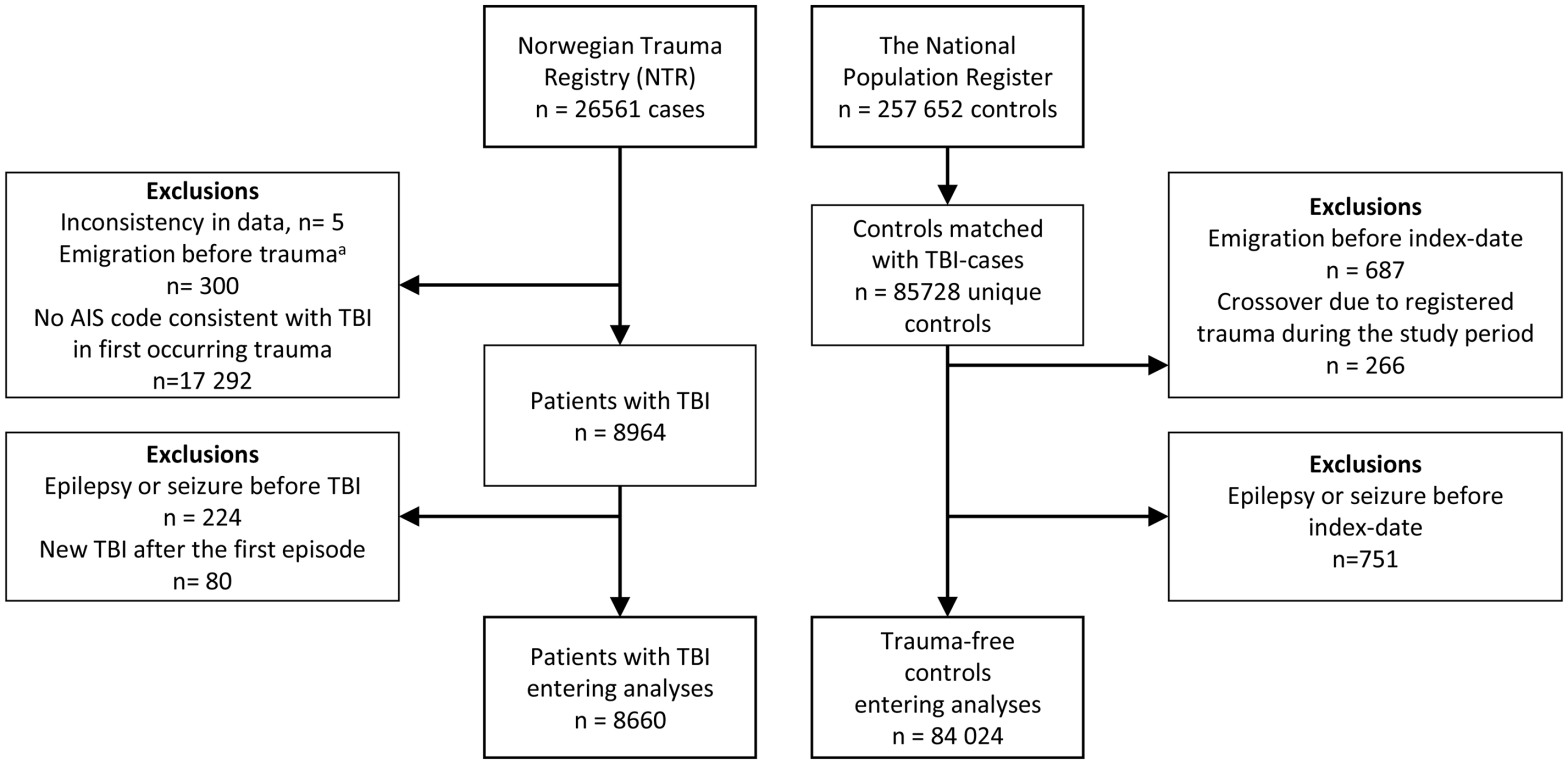
Overview of the study population. TBI, Traumatic brain injury. AIS, Abbreviated Injury Scale. ^a^Emigrated Norwegians experiencing trauma when visiting Norway.

We excluded patients and controls who had a diagnosis of epilepsy or seizures during the last year before the index date. This was defined as health care contact classified with ICD-10 codes G40, G41 or R56.8 in the NPR, or dispense of a prescription under the reimbursement codes for epilepsy in the NorPD. This includes ICD-10 code G40 and International Classification of Primary Care, 2nd edition (ICPC-2) code N88. Patients with one or more subsequent TBIs after the first trauma were excluded to avoid complicating any possible causal relationship between exposure (first TBI) and outcome (epilepsy). Overview of the study population is presented in Figure 1.

### 2.3 Data collection and definitions

Demographic variables, preinjury health status, trauma event details, AIS codes and Injury Severity Scores (ISS) were extracted from the NTR. ICD-10 codes were obtained from the NPR and information on dispensed drugs was obtained from NorPD. Statistics Norway provided information on emigration and deaths.

Preinjury health was categorized according to the American Society of Anesthesiologists (ASA) Physical Status Classification System (23). In addition, we included a variable available from the NTR labeled “GOS” describing preinjury functional status, categorized in the same way as in the Glasgow Outcome Scale (GOS), i.e., a score of 5 represents good function with no disabilities (24). The main mechanisms of injury were divided into traffic-related accidents, low-energy falls (from a height ≤ 1 meter), high-energy falls (from >1 meter) and “other,” including gunshot wounds and injuries caused by stabbing, explosions, or blunt objects.

We categorized the severity of TBI based on AIS severity scores. If a patient had several AIS codes registered, the highest severity score was used. A score of 3 to 6 defined a moderate to severe TBI. In the stratified analysis, we divided AIS into three categories: mild (AIS 1–2), moderate (3–4) and severe (5–6). As another measure of TBI severity, the Glasgow Coma Scale was applied, stratified on mild (GCS 13–15), moderate (9–12) and severe (3–8). GCS was estimated upon admission. In patients where prehospital intubation had been performed, the prehospital GCS was used. Overall trauma severity was described by ISS. ISS is calculated based on AIS severity in the three most injured body regions (25). ISS >15 defined major trauma. We categorized types of TBI based on AIS codes listed in Supplementary Table 1. All categories except concussive injury reflect neuroimaging findings of TBI.

The primary outcome was epilepsy after TBI, consistent with PTE. Epilepsy was defined as health care contact with ICD-10 code G40 in the NPR or dispense of an anticonvulsive drug (Anatomical Therapeutic Chemical codes N03 and N05) under the reimbursement codes for epilepsy in the NorPD, hereafter referred to as anti-seizure medication (ASM). Onset of epilepsy was set at the date of the first health care contact with ICD-10 code G40, or at the date of the first dispense of ASM, whichever came first. Seizures within the first week after TBI were excluded according to the current definition of PTE (26). We also excluded dispense of pregabalin from the definition of epilepsy as it is primarily used for treating neuropathic pain and generally not used for epilepsy treatment in Norway. The same definitions were applied for the control group.

### 2.4 Statistical analysis

Patient and injury characteristics are presented as means with SD and frequencies (n) with percentages when appropriate. The cumulative incidence of epilepsy in the TBI group and the control group was estimated using Aalen-Johansen estimators, also allowing death as a competing event (27). Event times were calculated as days from date of TBI (index date) to date of the first occurrence of epilepsy, death, or censoring (at emigration or end of study, 31 December 2020). Cause-specific cumulative incidence curves with 95% pointwise confidence intervals (CIs) for epilepsy and death are presented for the overall TBI population and for subgroups defined by brain injury severity and age. The cumulative incidence is an estimate of the absolute risk and is also referred to as “risk” throughout the paper. Cumulative incidence ratios were calculated and referred to as “relative risk.”

Emigration may not be independent of outcome, as less injured patients may be more likely to emigrate than patients with severe TBI, which in turn carries a higher risk of PTE. To account for this, we performed a sensitivity analysis with emigration as a second competing event.

The occurrence and co-existence of different injury categories in patients with moderate to severe TBI (AIS ≥3) are visualized using UpSet plots (28, 29), including frequency (n with percentage) of PTE within the most frequently occurring injury types and patterns (n ≥ 10). Statistical analyses were performed using Stata version 16.0, R version 4.1.2 and RStudio version 2023.03.1, applying the R-packages “tidyverse,” “survival,” “lubridate,” “splitstackshape,” “ggsurvfit” and “ComplexUpset” (30).

## 3 Results

Of the 26,561 trauma-patients in the NTR study cohort, we identified 8,964 patients with TBI in the first occurring trauma. After excluding patients and controls with a history of epilepsy or seizures and patients with subsequent TBI, the final study population consisted of 8,660 patients with TBI and 84,024 trauma-free controls. Overview of the study population is presented in Figure 1.

Of the patients with TBI, 66% were males. The mean age at TBI was 41.6 years (SD =25.5, range 0–101), with a peak around 20 years of age. The majority were healthy (ASA score 1) with no or minor functional disability (GOS 5) prior to TBI. Twenty-four percent had major trauma and 35% had moderate to severe TBI (AIS ≥3), of these 99% had neuroimaging evidence of intracranial injury or skull fracture. Of the 5,631 (65%) patients with mild TBI (AIS 1–2), only 9.5% had AIS code(s) reflecting neuroimaging abnormality. The mean age in the control group was 41.1 years (SD = 25.5) and 66% were males.

For 1,073 (12%) of the patients with TBI and 4,716 (5.6%) of the controls, follow-up was stopped before the end of the study due to emigration (*n* = 99 in the TBI- group and *n* = 803 in the control group) or death (*n* = 974 in the TBI group and *n* = 3,913 in the control group).

During the study period, 321 (3.7%) of the patients with TBI and 347 (0.4%) of the controls developed epilepsy. Within the TBI group, patients who developed epilepsy were older and had more comorbidities, indicated by higher ASA-scores, than patients without epilepsy. They had more severe TBI both when classified using AIS and GCS. The most common injury mechanism in patients with epilepsy was low energy falls, while traffic accidents were more frequent in patients without epilepsy. Baseline and injury characteristics for the patients with TBI are presented in Table 1.

**Table 1:** Demographic, clinical and injury characteristics of patients with TBI.

	No epilepsy *n* = 8,339	Epilepsy *n* = 321
Age, years (mean, SD)	41.2 (25.5)	51.0 (24.2)
Sex		
Female	2,838 (34%)	91 (28%)
Male	5,501 (66%)	230 (72%)
Preinjury ASA score^a^		
ASA 1–2	7,262 (89%)	237 (76%)
ASA ≥ 3	884 (11%)	75 (24%)
Preinjury GOS^a^		
GOS 5	7,516 (92%)	271 (87%)
GOS ≤ 4	635 (7.8%)	42 (13%)
Injury mechanism^a^		
Traffic accidents	3,216 (40%)	72 (24%)
Low energy falls	1730 (21%)	114 (38%)
High energy falls	2,101 (26%)	75 (25%)
Other	1,067 (13%)	43 (14%)
AIS severity score		
AIS 1	3,142 (38%)	39 (12%)
AIS 2	2,391 (29%)	59 (18%)
AIS 3	1,631 (20%)	82 (26%)
AIS 4	531 (6.4%)	48 (15%)
AIS 5	635 (7.6%)	93 (29%)
AIS 6	9 (0.1%)	0 (0%)
Injury Severity Score (ISS)		
ISS > 15	1882 (23%)	166 (52%)
Glasgow Coma Scale score^a^		
13–15	6,926 (84%)	186 (59%)
9–12	536 (6.5%)	50 (16%)
3–8	792 (9.6%)	79 (25%)
Injury types^b^		
Penetrating injury	24 (0.3%)	<5 (<1.6%)
Subdural hematoma	1,442 (17%)	150 (47%)
Cerebral contusion / hematoma	1,428 (17%)	143 (45%)
Skull fracture	1745 (21%)	134 (42%)
Subarachnoid hemorrhage	1,445 (17%)	132 (41%)
Epidural hematoma	373 (4.5%)	34 (11%)
Cerebral edema	175 (2.1%)	35 (11%)
Concussive injury except DAI	5,306 (64%)	98 (31%)
DAI	168 (2%)	26 (8.1%)
Other injury	576 (6.9%)	51 (16%)
Death		
Died during study period	975 (12%)	47 (15%)
Death in study period		

ASA, American Society of Anesthesiologists Physical Status Classification System. GOS, Glasgow Outcome Scale. AIS, Abbreviated Injury Scale. NA, not applicable. DAI, diffuse axonal injury. ^a^Missing data: ASA: 202, GOS: 196, Injury mechanism: 242, GCS: 91. ^b^Several patients experienced more than one type of injury, so the sum of percentages exceeds 100.

### 3.1 Incidence of epilepsy

**Figure 2:**
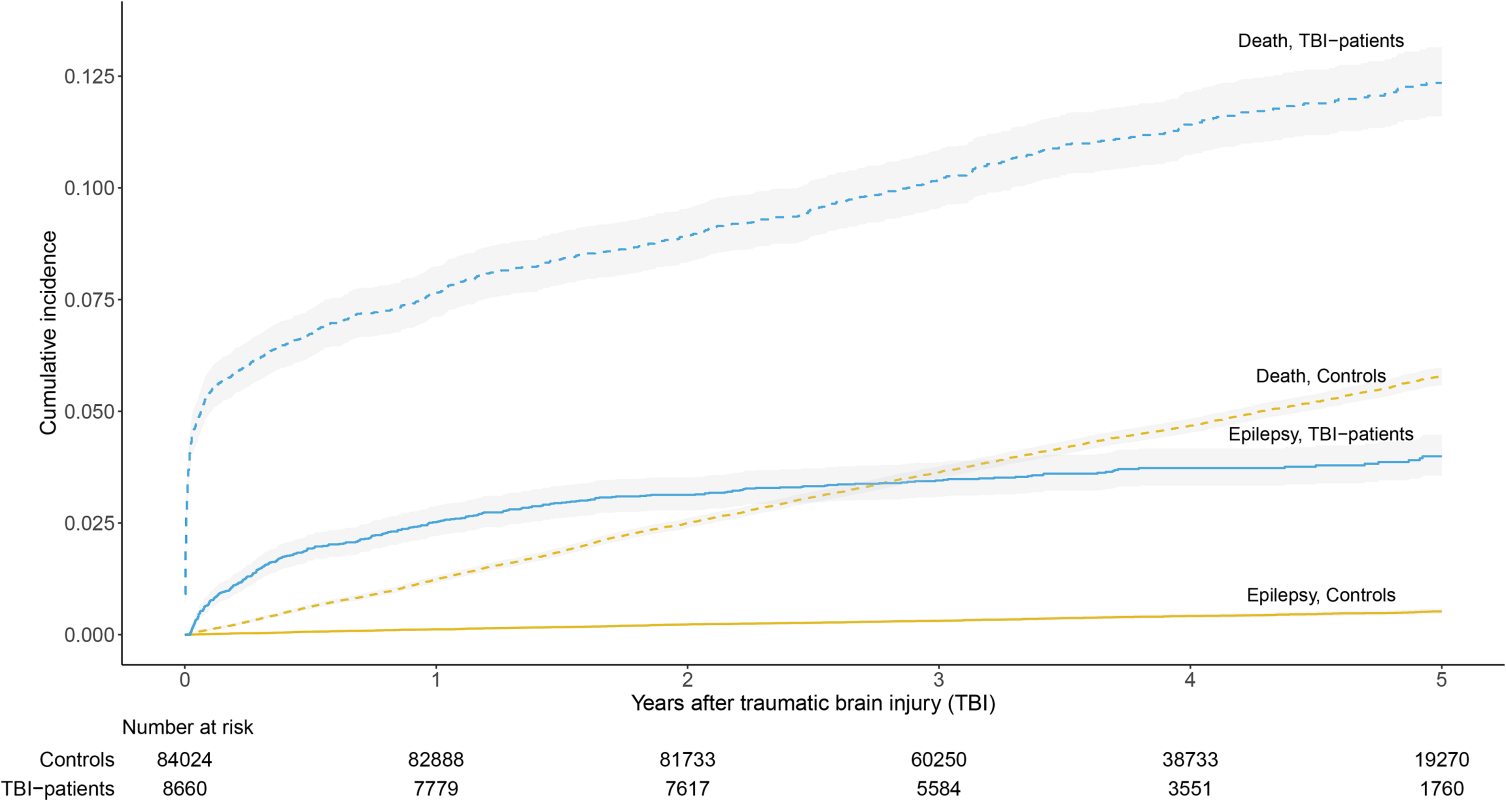
Cumulative incidence estimates of epilepsy and death in patients with TBI and trauma-free controls.

The cumulative incidence of epilepsy in patients with TBI was 3.1% (95% CI 2.8–3.5%) after 2 years and 4.0% (3.6–4.5%) after 5 years. The cumulative incidences in the control group were 0.2% (95% CI 0.2–0.3%) and 0.5% (0.5–0.6%), respectively (Figure 2). Compared to trauma-free controls, the relative risk of developing epilepsy was 13.6 (95% CI 11.3–16.4) during the first 2 years and 7.7 (6.5–9.0) during the first 5 years after TBI. The relative risk of PTE was highest the first 6 months after TBI, and 84% had onset of epilepsy within 2 years of injury. In a sensitivity analysis, treating emigration as a second competing event did not contribute to any meaningful change in the overall results.

**Figure 3:**
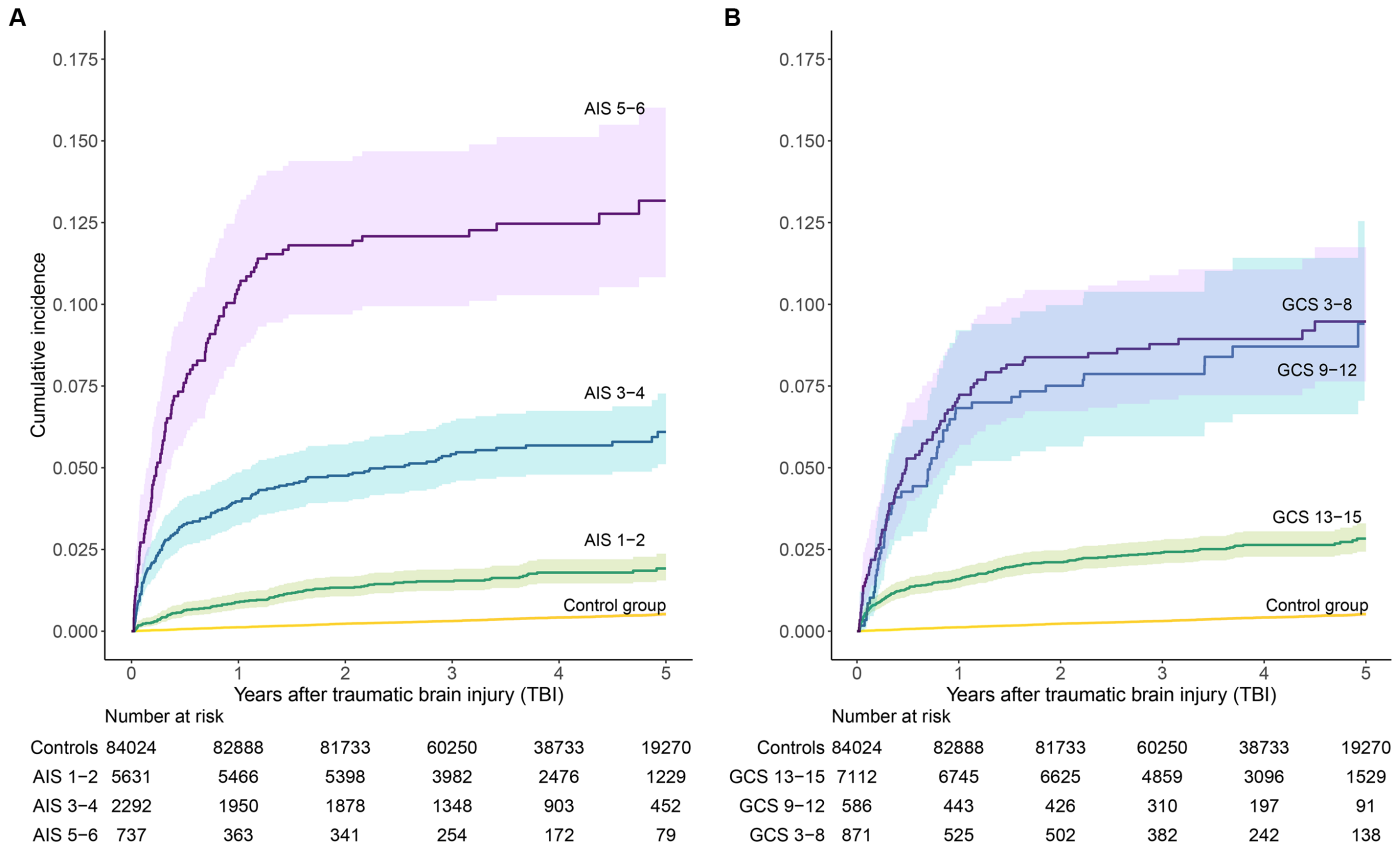
Cumulative incidence estimates of epilepsy. Patients with TBI are stratified by brain injury severity, **(A)** according to Abbreviated Injury Scale (AIS) and **(B)** according to Glasgow Coma Scale (GCS).

The risk of developing PTE increased with increasing brain injury severity, both when stratifying on AIS and GCS. When categorizing severity according to AIS, the 2- and 5-year cumulative incidences of PTE after severe TBI were 11.8% (95% CI 9.7–14.4%) and 13.2% (10.8–16.0%), respectively (Figure 3A). Using GCS, the cumulative incidence estimates were 8.4% (95% CI 6.7–10.4%) and 9.5% (7.6–11.7%; Figure 3B). For GCS, there were no significant differences in risk in patients with moderate TBI compared to those with severe TBI. Otherwise, differences in risk were significant between all groups (Figures 3A,B). Figures showing the cumulative incidences of death are provided in the Supplementary Figures 1, 2. Compared to trauma-free controls, the relative risk of developing epilepsy during the first 5 years was 25.3 (95% CI 20.1–31.7) among patients with severe TBI, 11.7 (9.5–14.4) in those with moderate TBI and 3.7 (2.9–4.7) in patients with mild TBI (severity categorized according to AIS).

**Figure 4:**
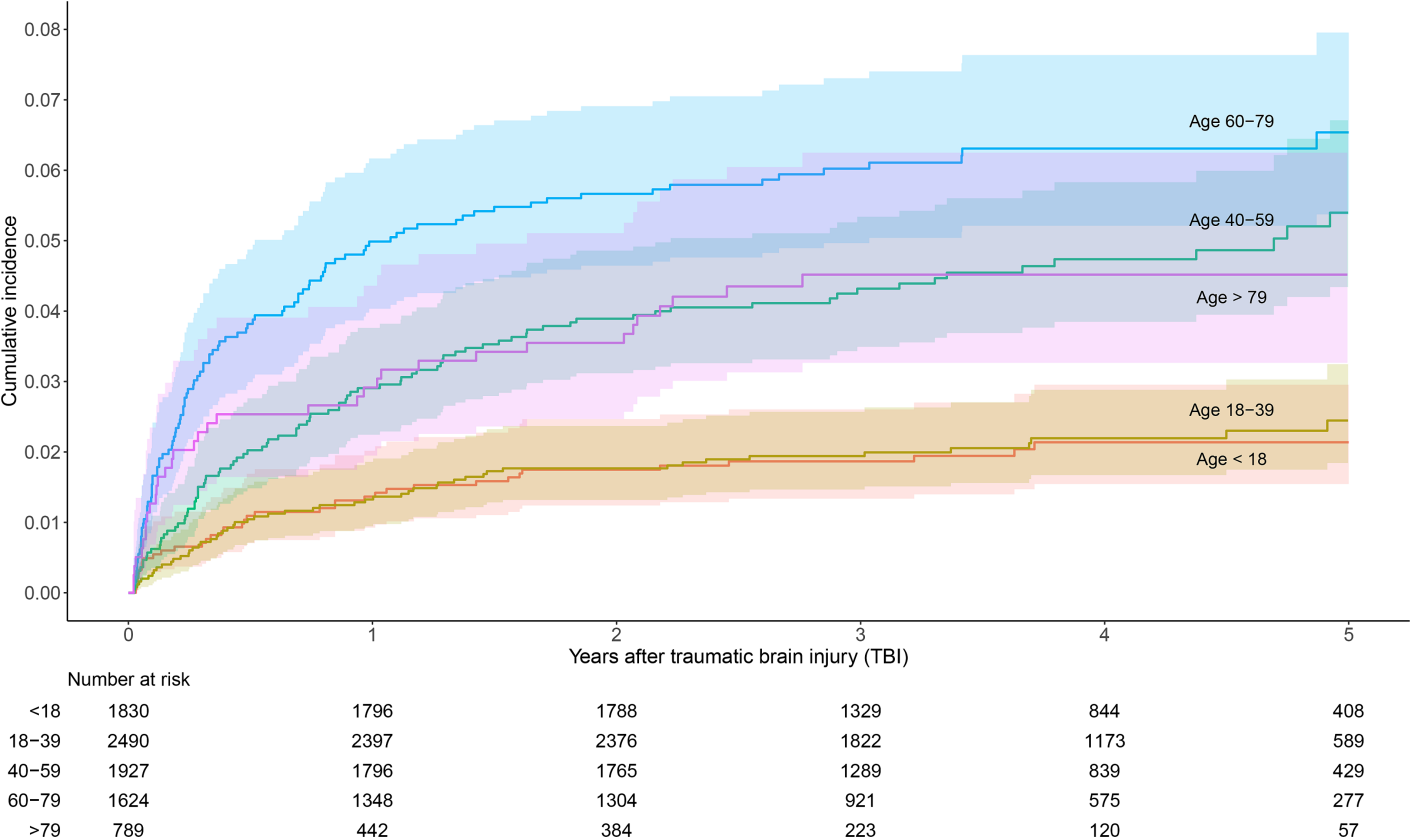
Cumulative incidence estimates of epilepsy in patients with TBI, stratified by age groups.

When stratifying patients with TBI by age groups, we found that the risk was highest among patients between 60 and 79 years, with a cumulative incidence of 5.7% (95% CI 4.7–6.9%) at 2 years and 6.5% (5.4–8.0%) at 5 years. The risk was significantly higher than the risk in patients aged 18–39 and in those <18 years, but the differences in risk between the older age groups were not significant (Figure 4). The cumulative incidence of death in patients aged +79 was markedly higher than in the other age groups (Supplementary Figure 3). Compared to the youngest patients (<18 years), the relative risk of PTE in patients aged 60–79 was 3.1 (95% CI 2.1–4.5) after 5 years.

### 3.2 Injury types and risk of epilepsy

**Figure 5:**
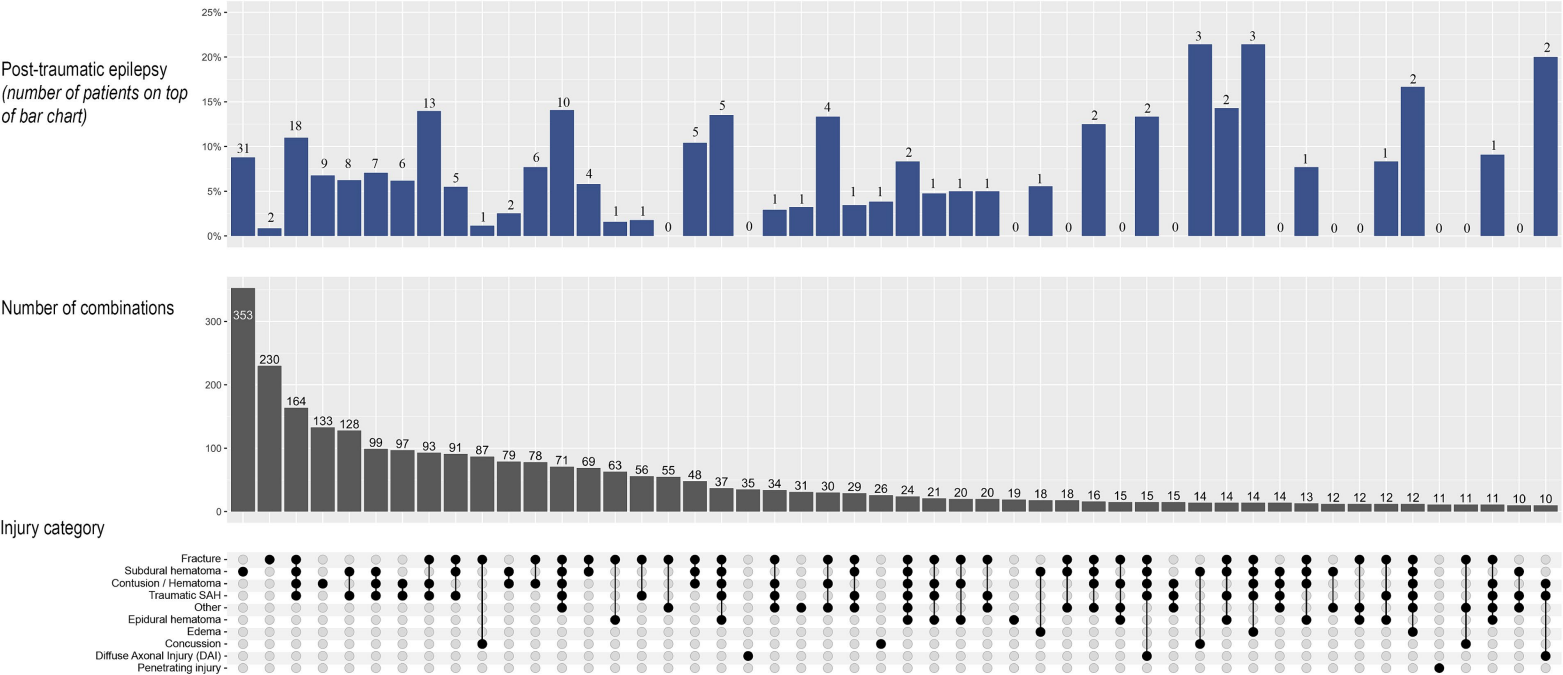
UpSet plot of injury types and occurrence of post-traumatic epilepsy (PTE) in patients with moderate to severe TBI (*n* = 3,029). In the lower section, the connecting dots illustrates occurrence and combinations of injury types. In the mid-section, the vertical bars depict the number of patients with the corresponding injury type or combination of injuries. In the upper section, the bars depict the frequency of PTE in the corresponding patterns, with percentage on the y-axis and number of patients on top of the bars. Injury types and combinations that occurred in less than 10 patients are not shown. SAH, subarachnoid hemorrhage.

In 90 (28%) of the patients developing PTE, there were no AIS codes reflecting neuroimaging evidence of intracranial injury or skull fracture. In the remaining 231 (72%) patients with PTE, the most frequent injury types were subdural hematoma (present in 47% of the patients), followed by cerebral contusion and/or hematoma and skull fracture (present in 45 and 42%, respectively). Except concussive injury, all injury types occurred more frequently in patients developing PTE than in patients without epilepsy (Table 1). Most of the patients with moderate to severe TBI (AIS ≥3) had multiple injury types reflected by several AIS codes defining TBI (range 1–9). The occurrence and co-existence of injury types and the prevalence of PTE in different injury types and patterns are illustrated in Figure 5. In general, the highest prevalence was seen in patients with multiple injuries. Of the isolated injuries, PTE occurred most frequently in patients with subarachnoid hemorrhage (28.6% among *n* = 7), followed by subdural hematoma (8.8%) and cerebral contusion/hematoma (6.8%), whereas only 0.9% of the patients with isolated skull fracture developed PTE.

## 4 Discussion

In this nationwide matched cohort study, we found a 2- and 5-year cumulative incidence of epilepsy of 3.1 and 4.0% in hospitalized patients with TBI. Compared with trauma-free controls, patients with TBI had 7.7 times the risk of developing epilepsy during the first 5 years after TBI. Brain injury severity and age were important prognostic factors.

The identification of a high risk of epilepsy after TBI that increases with increasing brain injury severity, and that remains increased for at least 5 years after injury, is consistent with previous findings (2, 4). Several groups have studied the epidemiology of post-traumatic seizures during the last decades, and the reported incidence of PTE varies between approximately 2 and 25% (31, 32). Variability in findings may be related to heterogeneity in populations being studied, definitions of TBI severity, length of follow-up and statistical methods used. In a population-based study including patients with TBI in the period 1935–1984, the estimated 5-year cumulative incidence of epilepsy was 0.7% after mild TBI, 1.2% after moderate TBI and 10.0% after severe TBI (2). While our results regarding those with severe TBI are similar, we found a higher risk in those with mild and moderate TBI. Different from this study, we included only hospitalized patients, possibly implying a higher level of severity within these groups. Also, the proportion of elderly (+65 years) in our TBI- population was higher (22.9% vs. 5.9%), reflecting the current demography of TBI (1).

More recent studies have reported notably higher risks of PTE compared to our findings (5, 12, 14, 33). This may be related to differences in study population, as these studies included selected groups of patients with moderate–severe TBI, in contrary to ours, which was a nationwide population-based study. Differences in study design, i.e., prospective observational studies with self-report or clinical follow-up versus register-based studies, may also contribute to disparate study findings. There is a possibility that use of statistical methods censoring on death may have led to inflated estimates of the risk of PTE in previous studies (34). This bias will affect groups with a high mortality most, notably patients with severe TBI and those with the highest age. In the present study, we plotted cumulative incidence of death alongside epilepsy to illustrate and better evaluate how the competing risk from death may influence epilepsy incidence.

Consistent with other studies, we found that more than 80% of the patients developing PTE did so within 2 years after TBI (12, 33). Due to a possible delay from seizure onset to epilepsy diagnosis, the proportion of patients developing epilepsy before 2 years post-injury may be even higher (35).

We evaluated two ways of categorizing injury severity; one based on neuroimaging findings (AIS), and one based on assessment of the level of consciousness (GCS). GCS is widely used for measuring brain injury severity but may be highly influenced by use of alcohol and sedatives or metabolic derangements. We found a higher risk of PTE in patients with severe injury according to AIS compared to GCS, indicating that lesion volume is a better predictor than GCS for selecting patients at high risk of PTE.

Similar to previous studies, we a found higher risk of PTE in older patients compared to the younger age groups (2, 15), which may suggest an increasing susceptibility to epilepsy with higher age. However, we found no significant difference in cumulative incidence of PTE between patients aged 40–59 years and the older patient groups. The apparently lower risk in the oldest patients (>79 years) compared to the 60–79 years olds may be explained by the markedly higher risk of death in the >79-group. This leads to a more rapid fall in the number at risk, which may result in a lower cumulative incidence (27). Also, there is a possibility that seizures to a greater extent may be overseen or misdiagnosed in the elderly (36).

Structural injuries such as skull fracture, brain contusion or parenchymal hemorrhage, subdural hematoma and dural penetration have previously been identified as significant risk factors for PTE (12, 13). Our results illustrate that patients with moderate to severe TBI often have a complex pattern of injuries (Figure 5). This complicates stratification of patients into different risk groups based on injury type, since associations between specific injury types and risk of epilepsy may be confounded by other injuries. Our findings suggest that this is particularly evident in skull fractures: 42% of the patients with PTE had a skull fracture, but only 0.9% of the patients with moderate to severe TBI and a skull-fracture-only injury developed epilepsy. In contrary to this, a high frequency of PTE was seen in patients with moderate to severe TBI and isolated subarachnoid hemorrhage. This finding is probably not representative for subarachnoid hemorrhages in general, but merely related to the very small number of patients in his group.

Early post-traumatic seizures have been associated with an increased risk of PTE (12, 14). Information about early seizures was not available from the NPR or the other registries, so we were not able to include this in the analyses. Prophylactic treatment with ASM to prevent early seizures is not in routine use in Norway, as it has not been found to be effective in preventing PTE (37, 38). The lower incidence of PTE in our study compared to studies with patients receiving prophylactic drugs supports this finding.

Our results are in line with previous reports that shows that the risk of developing epilepsy is increased also after mild TBI (4). Almost one third of the patients developing PTE in our study had mild TBI, and > 90% of these patients had no CT scan findings related to TBI. This indicates that risk factors other than injury severity and structural damage influence the outcome. In addition to age and comorbidity, genetic variability may play an important role in the process of epileptogenesis (39). It is worth mentioning that most people with mild TBI will not be treated in-hospital. Patients with mild TBI in our study are trauma-patients that may also have suffered substantial extra-cranial injury or have other characteristics that may increase the risk of epilepsy. Due to this selection bias, our results regarding incidence of epilepsy in mild TBI may not be generalizable to all mild TBIs.

Norway is a high-income country with universal access to high-quality healthcare and reimbursement of expenses related to treatment of most medical conditions. A high standard of TBI care may affect the outcomes after TBI including the incidence of PTE. However, our results should be representable for other high-income countries. The lower risk of PTE found in our study and similar studies from the other Scandinavian countries (3, 15) compared to studies performed in the United States (5, 33) raises the question if there indeed are geographic variations or genetic differences in PTE susceptibility, or if the discrepancy mainly is due to diagnostics, selections in the patient recruitment and other methodological differences.

There are limitations in using registry data for evaluation of epilepsy diagnosis. Misclassification of epilepsy may occur if diagnostic coding is not correct. Recategorization of the epilepsy population to include only those with ≥2 registrations of an epilepsy diagnosis would increase the positive predictive value of the diagnosis, but also exclude a substantial number of patients with epilepsy (40). To increase the likelihood of identifying all individuals with epilepsy we included use of ASM in the definition of the diagnosis. The use of reimbursement codes minimizes the possibility that individuals received ASMs for other indications than epilepsy. However, prescription of ASMs for short-term prophylaxis after early post-traumatic seizures may have led to an overestimation of PTE during the first months after TBI. There is also a possibility that an epileptic seizure in a previously undiagnosed patient may cause TBI, and hence overestimate the incidence of PTE.

Due to the study design, 5 years of follow-up was not available for all participants. As a result, the estimates of the 5-year incidence of epilepsy are less precise than those based on the 2-year follow-up period. Another limitation with the design of this study is the short observation period before trauma. Thus, there is a possibility that prior seizures and diagnoses of epilepsy may have been missed in the exclusion process. Since patients with epilepsy have an increased risk of TBI compared with controls (41), this might have contributed to an overestimation of epilepsy in patients with TBI versus the control group. Despite these limitations, the use of extensive nationwide registers resulting in a large sample size representing the total population of hospital-admitted patients with TBI, the use of a matched control group and the competing risk analysis, accounting for the increased risk of death among patients with TBI, ensure valid and up-to-date estimates of the cumulative incidence of PTE.

In summary, we found that patients with TBI have a high risk of developing epilepsy compared to trauma-free controls, and that the risk increases with brain injury severity and age. The lower risk in patients with moderate–severe TBI in our study compared to previous reports raises the question if there are geographic or genetic variations in PTE susceptibility.

## Data availability statement

The data analyzed in this study is subject to the following licenses/restrictions: data may be obtained from a third party and are not publicly available. The data were used under license for the current study. Projects with necessary approvals and legal basis according to the EU General Data Protection Regulation may request data sharing/case pooling. Requests should be directed to principal investigator LR, lrossela@ous-hf.no.

## Ethics statement

The studies involving humans were approved by Regional Committee for Medical Research Ethics (REC) South East (Reference number 2018/2010). A Data Protection Impact Assessment was conducted in cooperation with the Data Protection Officer at Oslo University Hospital. The studies were conducted in accordance with the local legislation and institutional requirements. REC waived the requirement of written informed consent for participation from the participants or the participants’ legal guardians/next of kin because the benefit to society was considered to outweigh the potential harm for the individual by being included in the study.

## Author contributions

HS: Conceptualization, Formal analysis, Methodology, Writing – original draft, Writing – review & editing, Visualization. TN: Data curation, Formal analysis, Methodology, Visualization, Writing – review & editing. AR: Writing – review & editing. LR: Conceptualization, Supervision, Writing – review & editing. JS: Formal analysis, Methodology, Writing – review & editing. JG: Formal analysis, Methodology, Writing – review & editing. EH: Supervision, Writing – review & editing. ET: Conceptualization, Supervision, Writing – review & editing.
