# Supplementary material for: Risk of epilepsy after traumatic brain injury: a nationwide Norwegian matched cohort study
Source: Front Neurol. 2024 Jun 5;15:1411692. doi: 10.3389/fneur.2024.1411692 (PMC11188468; doi:10.3389/fneur.2024.1411692)
Supplement: Supplementary file 1 [file Data_Sheet_1.PDF]

Supplementary Material

Supplementary Table 1. Overview of codes defining traumatic brain injury and categorization of the codes into injury type categories.

INJURY CATEGORY	AIS CODES INCLUDED IN CATEGORY[a]
Cerebral contusion and/or intracerebral hematoma	140602 - 140624, 140626, 140638 - 140649
Penetrating injury	116000 - 116004, 140216, 140690 - 140692
Skull fracture	150000 - 150408
Epidural hematoma	140630 - 140636
Subarachnoid hemorrhage	140693 - 140698
Brain edema	140660 - 140674
Diffuse axonal injury (DAI)	140625, 140627 - 140628, 161007 - 161013
Concussive injury (except DAI)	160000 - 161006
Other brain injury related to trauma	113000, 140629, 140675 – 140688, 140699

[a]Abbreviated Injury Scale (AIS) 2005, update 2008

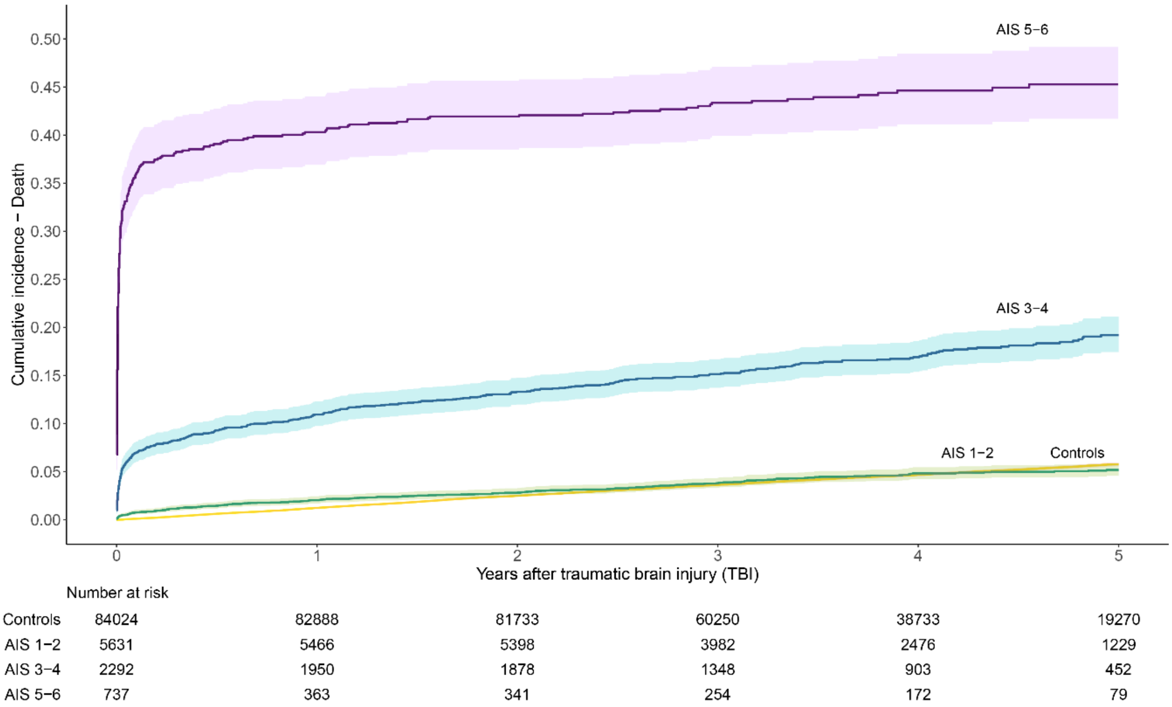

Supplementary Figure 1. Cumulative incidence estimates of death. Patients with TBI are stratified by brain injury severity according to Abbreviated Injury Scale (AIS). Note the difference in the y-axis compared to Figure 3A.

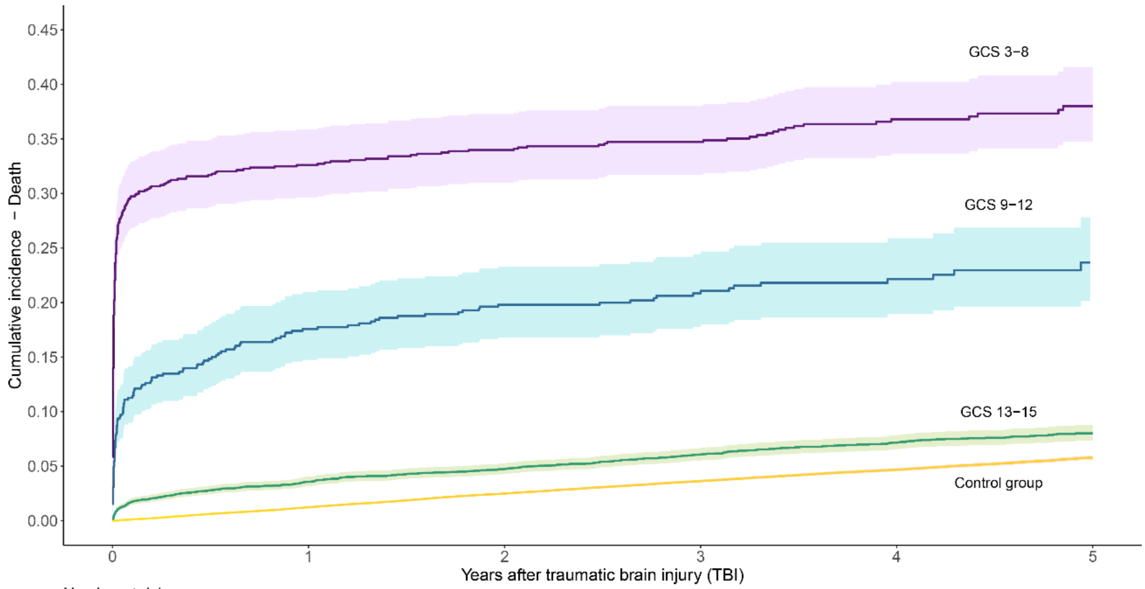

Number at risk

	0	1	2	3	4	5
Controls	84024	82888	81733	60250	38733	19270
GCS 13−15	7112	6745	6625	4859	3096	1529
GCS 9−12	586	443	426	310	197	91
GCS 3−8	871	525	502	382	242	138

Supplementary Figure 2. Cumulative incidence estimates of death. Patients with TBI are stratified by brain injury severity according to Glasgow Come Scale (GCS). Note the difference in the y-axis compared to Figure 3B.

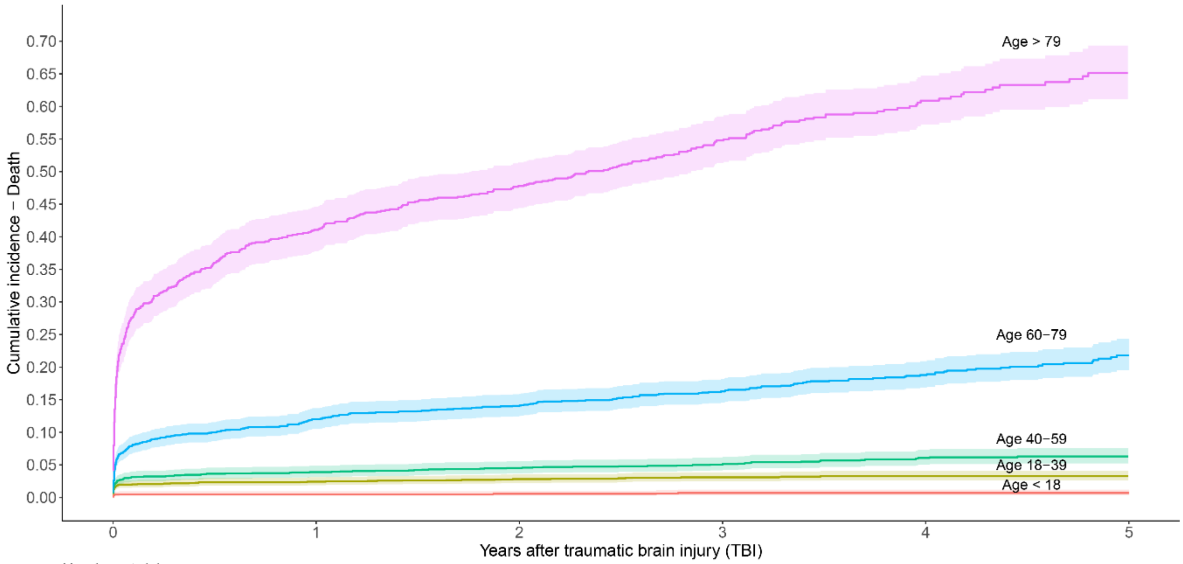

Number at risk

	0	1	2	3	4	5
<18	1830	1796	1788	1329	844	408
18−39	2490	2397	2376	1822	1173	589
40−59	1927	1796	1765	1289	839	429
60−79	1624	1348	1304	921	575	277
>79	789	442	384	223	120	57

Supplementary Figure 3. Cumulative incidence estimates of death in patients with TBI, stratified by age groups. Note the difference in the y-axis compared to Figure 4.
